# Pilot Study on the Effect of Biophysical Therapy on Salivary Alpha-Amylase as a Surrogate Measure of Anxiety/Stress: In Search of a Novel Noninvasive Molecular Approach for the Management of Stress

**DOI:** 10.3390/ijms21020415

**Published:** 2020-01-09

**Authors:** Ida Ferrara, Colin Gerard Egan, Alberto Foletti

**Affiliations:** 1 Studio Medico Gunè, 80011 Acerra, Italy; idaferrara@gmail.com; 2 Clinical Biophysics International Research Group, 6900 Lugano, Switzerland; 3 CE Medical Writing, 56023 Pisa, Italy; colingegan@gmail.com; 4 Institute of Translational Pharmacology, National Research Council-C.N.R., 00185 Rome, Italy

**Keywords:** anxiety, work stress, salivary alpha-amylase, biophysical therapy, electromagnetic information transfer

## Abstract

Anxiety and depression impact dramatically on public health, underlying the importance of alternative cost-effective treatments. Previous studies have shown that biophysical treatment can significantly reduce anxiety symptoms and recently, salivary alpha-amylase (SAA) has been identified as an objective correlate of the sympathetic-parasympathetic imbalance related to increased stress burden, defined as allostatic load. The aim of this study was to evaluate the effect of biophysical therapy on SAA levels, in addition to the Depression Anxiety Stress Scale (DASS)-21 questionnaire. Twenty-four workers (sales representatives) presenting with mild anxiety/stress symptoms (Generalized Anxiety Disorder 7-item scale of > 5) were randomized to biophysical treatment (*N* = 12) or placebo control (*N* = 12). The biophysical group underwent electromagnetic information transfer through an aqueous system procedure, with daily self-administration for one month. SAA collection and the DASS-21 questionnaire were undertaken at baseline and after one month in all patients. Clinical characteristics and baseline DASS-21 subscale scores were similar between placebo and biophysical group at baseline. After one month, patients receiving biophysical therapy had significantly reduced SAA levels compared to the placebo group (27.8 ± 39.4 vs. 116.8 ± 114.9 U/mL, *p* = 0.019). All three DASS-21 subscales, depression (9.3 ± 5.1 vs. 5.7 ± 5.5, *p* = 0.1), anxiety (6.7 ± 25 vs. 3.7 ± 2.2, *p* = 0.0049) and stress (10.8 ± 4.2 vs. 7.3 ± 3.7, *p* = 0.041) were also decreased after biophysical treatment compared to placebo after one month. Our findings suggest that biophysical therapy can benefit workers with mild (subclinical) anxiety/stress. These results were also validated by the concomitant reduction of SAA levels and an improvement in DASS-21 subscales. The underlying molecular mechanisms of this therapy remain to be characterized.

## 1. Introduction

Stress burden is recognized as an important prognostic factor associated with increased morbidity and mortality [1,2]. The cumulative effect of stress burden leads to an impairment in allostatic homeostasis, giving rise to allostatic load [3,4,5,6]. Thus, interventions aimed at decreasing allostatic load could provide benefit in terms of decreasing morbidity and mortality [7]. It is recognised that the endogenous electromagnetic activity can play a key role in embryonic development, cell differentiation and during reparative processes [8]. Moreover, endogenous electrodynamic activity [9,10,11,12] appears to play a central role in the short and long-range hierarchical synchronization of physiological activity aimed to maintain allostasis and restore allostasis when allostatic load is altered.

The term electromagnetic homeostasis [13] has been suggested to describe these dynamics identifying a critical role of electromagnetic signalling in order to establish and tune the inner coherence of endogenous life rhythms [14]. The working hypotheses is that biophysical treatment can induce their clinical benefit through a resonance effect [15]. Resonance occurs between therapeutically delivered electromagnetic (endogenous or exogenous) signals to target tissues, organs, and/or the entire organism [16,17], resulting in both local and systemic effects [18]. To date, biophysical therapeutic methods have emerged as integrative tools in clinical practice and their potential beneficial use has been documented for the management of pain [19], in particular joint pain [20,21], back pain [22,23], neck pain [24], psoriasis [25], chronic kidney disease [26], and minor anxiety and depressive disorders [27].

Recently, salivary alpha-amylase (SAA) has been identified as an objective correlate of the sympathetic-parasympathetic imbalance related to increased stress burden, defined as allostatic load [28,29,30,31,32]. The aim of the present pilot study was to evaluate the effect of biophysical therapy on SAA levels in addition to its effect on the Depression Anxiety Stress Scale (DASS)-21 questionnaire.

## 2. Results

### 2.1. Baseline Clinical and Demographic Characteristics

A total of 24 subjects were included in the study, 12 (mean age: 40.1 ± 8.6 years) in the biophysical therapy group and 12 (mean age: 38.3 ± 5.6 years) in the control group. After completion of the Generalized Anxiety Disorder 7-item scale (GADS-7) questionnaire, physicians only included patients having a GAD-7 score of > 5, defined as having mild anxiety/depressive symptoms. Clinical characteristics and DASS-21 subscale values for control and biophysical patient groups are presented in Table 1. The majority of patients were female (20/24; 83.3%) and approximately half of patients were cigarette smokers (11/24; 45.8%). All patients completed secondary level education (high school) and about one-third attained third level (university) education (7/24; 29.2%).

### 2.2. Effect of Biophysical Treatment on Salivary Amylase Levels

**Figure 1:**
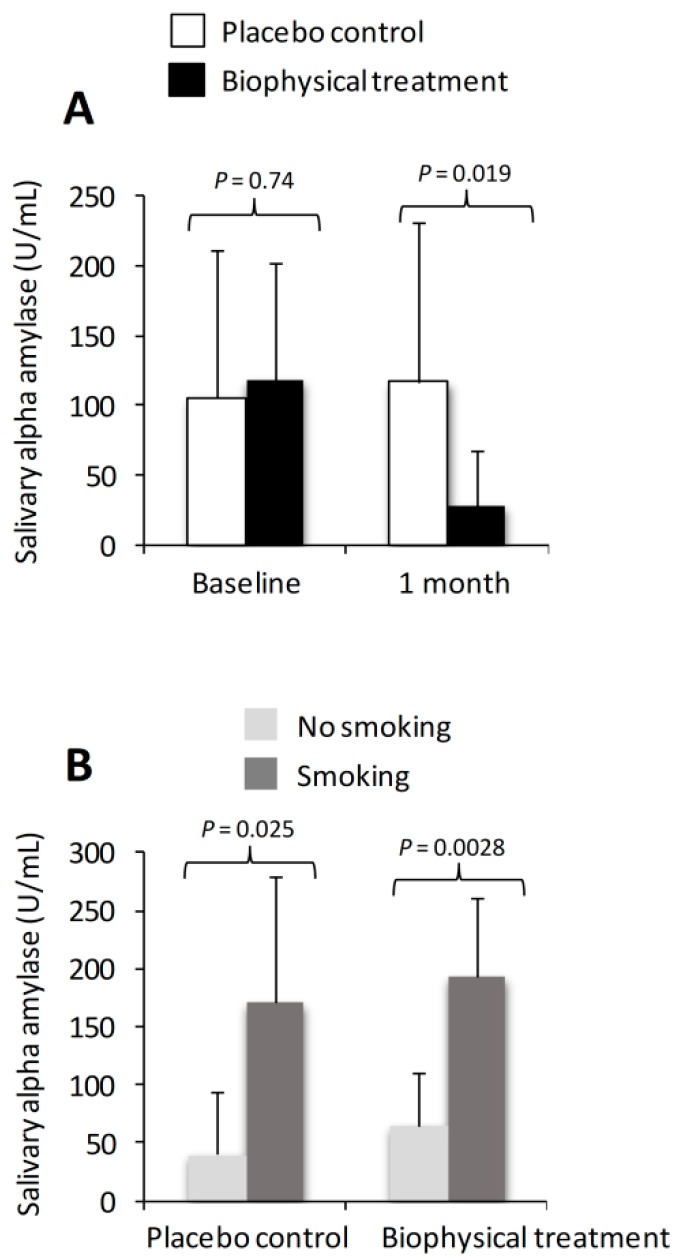
Effect of biophysical therapy and placebo control on salivary amylase levels (SAA). SAA levels in biophysical therapy and control groups at baseline and after one month (**A**) and SAA levels in patients who smoke compared to nonsmokers are shown (**B**). Data are presented as mean ± SD and *p*-values denote statistical significance between groups.

After one month of biophysical therapy, SAA levels were significantly decreased compared to the placebo control group (27.8 ± 39.4 vs. 116.8 ± 114.9 U/mL, *p* = 0.019) (Figure 1A). Exploratory sub-analysis revealed that in patients who smoked, levels of SAA were significantly higher at baseline compared to nonsmokers. This difference was observed to a similar extent in both control and biophysical groups (Figure 1B). Age and gender were not observed to influence SAA levels at baseline or follow up.

### 2.3. Effect of Biophysical Treatment DASS-21 Subscales

**Figure 2:**
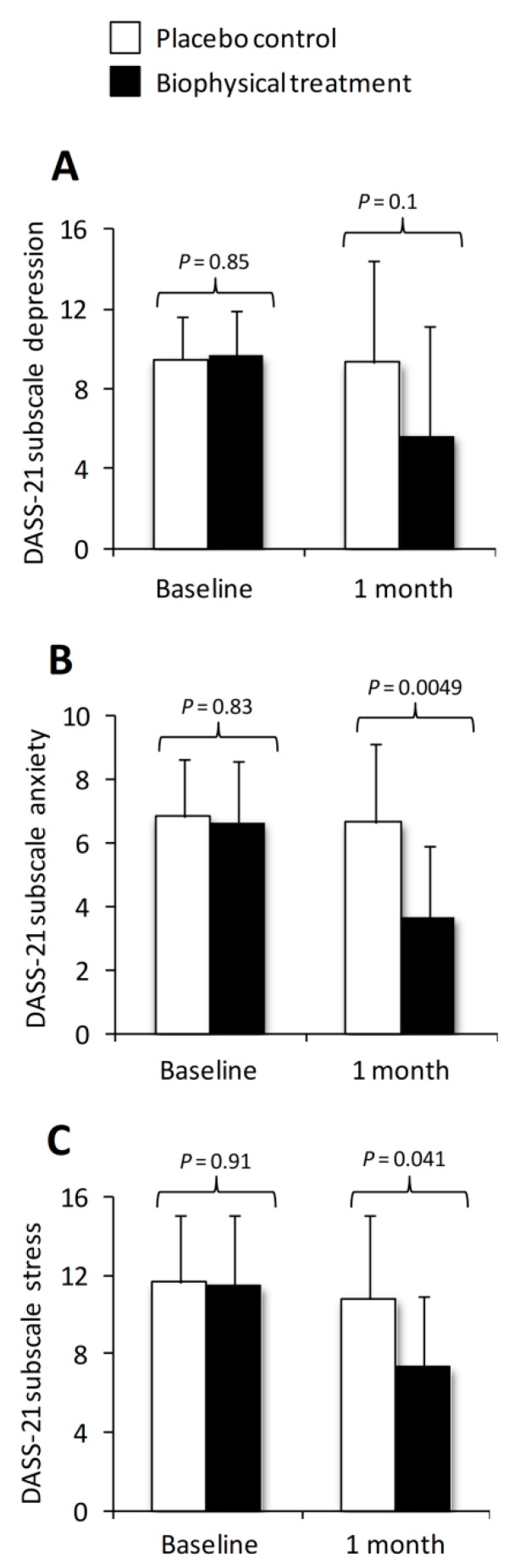
Effect of biophysical therapy and placebo control on DASS-21 subscales. The three subscales include depression (**A**), anxiety (**B**) and stress (**C**). Data are presented as mean ± SD and *p*-values denote statistical significance between groups.

All patients completed the self-reported DASS-21 questionnaire at baseline and after one month. In patients assigned biophysical therapy, a reduction in the depression subscale was observed compared to the control group, although this difference did not attain statistical significance (5.7 ± 5.5 vs. 9.33 ± 5.1, *p* = 0.1) (Figure 2A). However, comparing scores only within the biophysical group revealed a statistically significant reduction (5.7 ± 5.5 vs. 9.7 ± 2.2, *p* = 0.047). For the anxiety subscale, biophysical treatment resulted in a statistically significant improvement compared to placebo control group (6.7 ± 2.5 vs. 3.7 ± 2.2, *p* = 0.0049) (Figure 2B). A similar improvement was also observed for the third subscale, stress, where the biophysical therapy significantly decreased this subscale compared to the placebo control group (10.8 ± 4.2 vs. 7.3 ± 3.7, *p* = 0.041) (Figure 2C). No differences were observed between control and biophysical groups for the scores among the three subscales at baseline. Age, gender, or smoking status were not associated with DASS-21 subscale scores at baseline or follow up.

## 3. Discussion

The main findings that have emerged from this pilot study demonstrate that patients presenting with mild anxiety/depression (as judged by GADS-7 score of > 5) can experience a significant reduction in these symptoms as early as one month after biophysical therapy. Furthermore, by including SAA as an objective surrogate marker of anxiety/stress [28,29,30,31,32] in our study, this also allowed us to validate our results observed for DASS-21. Indeed, the observed improvement in patients following biophysical treatment resulted in a significant decrease in SAA levels, as well as a paralleled decrease in the three DASS-21 subscales depression, anxiety, and stress.

Psychological stress, anxiety, and depression impact dramatically on an individual’s quality of life [33], as well as impacting on healthcare resources, in terms of medical personnel and economic cost [34]. Furthermore, individuals with mild anxiety and depressive symptoms may even experience considerable burden in terms of quality of life, well-being, and work-related problems. A significant proportion of individuals suffer from anxiety/depression that can be categorised as “mild” and therefore are frequently not eligible for treatment using anxiolytic medication or anti-depressant drugs since this medication is either not indicated or not appropriate for patients with these milder symptoms. Furthermore, individuals with milder forms of anxiety disorders are less likely to seek treatment or specialist consultation. As a result, an increasing number of patients may fail to receive the appropriate care and frequently remain untreated. On this basis, there is an unmet need to provide alternative therapeutic options that alleviate these symptoms without many of the side effects associated with conventional psychoactive drugs.

In the recent years, biophysical therapy has been shown to exert beneficial effects in patients suffering from various types of pain [19,20,21,22,23,24], as well as in the management of psoriasis [25], early stages of chronic kidney disease [26], and more recently for minor anxiety and depressive disorders [27]. The precise mechanism by which biophysical treatment exerts its anxiolytic effects is poorly understood. However, preclinical studies suggest that it may be linked to a range of modulatory factors such as antioxidants, growth factors, and inflammatory mediators, although, it is an area that remains controversial [35,36]. Cichoń et al. in several studies have evaluated the effect of extremely low-frequency electromagnetic fields (ELF-EMF) on clinical outcome, as well as a range of biochemical and molecular measures in post stroke patients. After a four-week rehabilitation program where one group also received ELF-EMF field (40 Hz, 7 mT for 15 min/day for four weeks), ELF-EMF induced a significant improvement in functional (Activities of Daily Living) and mental (Mini-Mental State Examination and Geriatric Depression Scale) status. As well as an increase in antioxidant gene expression, nitric oxide metabolites, oxidative stress, and interleukin-1β were all improved/increased [37,38,39,40,41,42]. Indeed, a neuroprotective role of IL-1β-dependent in the regulation of neurotrophic factors has already been hypothesized [43].

Several in vivo studies have explored other potential molecular mechanisms associated with low-frequency electromagnetic waves on anxiety/depressive disorders. The potential mechanisms by which low-frequency electromagnetic waves exert their effects may occur via two mechanisms that are closely associated with each other; synaptic function and neural oscillation. It has been shown that theta phase coupling was positively correlated with synaptic plasticity in the ventral CA1 hippocampus region—medial prefrontal cortex (mPFC) pathway in depression rats [44]. In addition, Xu et al. (2013) reported that CA3-CA1 (hippocampus regions) synaptic plasticity was positively correlated with the unidirectional indices from CA3 to CA1 in melamine-treated rats [45].

Although the pathway from the ventral hippocampus to the mPFC is thought to play an important role in emotional memory processing, the CA3-CA1 pathway has also been found to be associated with spatial cognition [46,47]. It has also been observed that LFPMF (1 Hz) can increase the energy of low frequency bands (1–40 Hz) of human brain, especially the band of 1–3 Hz, termed “resonance effects” [48].

Yang et al. (2019), recently explored the potential modulatory effect of low-frequency pulsed magnetic field (LFPMF) on reversing cognitive impairment in a rat model of depression induced by chronic unpredictable stress [49]. After eight weeks of CUS, animals gained less weight but also exhibited anhedonia, anxiety, and cognitive decline in behavioural tests compared to controls. After two-week treatment of LFPMF (20 mT, 1 Hz magnetic stimulation), impairment of spatial cognition, as well as hippocampal synaptic function was reversed, including long-term potentiation and related protein expression. Thus, LFPMF has been shown to provide effective improvement on depressant behaviour and cognitive dysfunction in CUS rats, possibly via regulating synaptic function.

SAA has recently been seen to represent a surrogate marker of stress and anxiety [28,29,30,31,32]. It is also recognised that the sympathoadrenal medullary (SAM) is associated with anxiety and awakening [50] and it has been suggested that SAA may be used as an index of SAM activity since branches of sympathetic and parasympathetic nerves are distributed in the salivary glands. While the stimulation of sympathetic nerves can result in an increase in salivary protein secretion, the stimulation of parasympathetic nerves can increase saliva flow [51]. SAA activity is connected with the sympathetic nervous system stress response [52]. Thus, we can postulate that the effect of low electromagnetic waves (biophysical therapy) in our study may occur in part via regulation of synaptic function.

Indeed, in the present study, the inclusion of this marker as an endpoint serves two purposes: (a) To determine whether biophysical therapy can alter SAA levels in patients with mild anxiety/depressive symptoms and (b) to confirm any changes in SAA levels using a self-reported validated questionnaire for the assessment of anxiety/stress (DASS-21).

This pilot study revealed that even in individuals with “mild” anxiety/depressive symptoms, biophysical therapy administered for a period of one week could still afford a measurable improvement as evidenced by a significant reduction in both SAA and DASS-21 subscales. Interestingly, we also observed that in patients who smoked cigarettes, levels of SAA were significantly higher at baseline compared to nonsmokers. This difference was the same for both control and biophysical groups. Earlier studies have shown that cigarette smoking can cause an increase in both levels of serum and SAA activity [53,54,55]. Furthermore, acute administration of nicotine to nonsmokers has been associated with increased SAA [56]. The mechanism is thought to occur by nicotine decreasing the release of amylase from pancreatic cells (acini) in response to hormones (insulin and cholecystokinin) with a consequent increase in intracellular amylase [57]. Further studies examining the relationship between SAA and smoking and other co-morbid diseases is warranted.

It is important to highlight that since this was a randomized, double-blind, placebo-controlled trial, neither the patients nor the physician delivering the biophysical procedure were aware of the group assignment. Actually, all patients were treated with the same procedure and randomization was performed by the nurse at the time of delivery of the bottle of drops (Nomabit Base) according to the randomization string.

SAA is emerging as a reliable biomarker tool to assess allostatic load [28,29,30,31,32] allowing a single, reliable, and easy sampling. The added benefit of measuring SAA is that it provides an unbiased surrogate measure of anxiety/stress that cannot be influenced by the patient or physician. The biophysical treatment delivered through the electromagnetic information transfer through aqueous system procedure [58] seems to disclose a number of potentially new clinical applications, even in the field of stress-related diseases. The emerging working hypotheses are that biophysical therapy can exert a rebalance of the sympathetic-parasympathetic imbalance due to a systemic effect on stress burden.

This study has several limitations that need be addressed. Although the follow up period of one month was sufficient time to detect a significant change in SAA and DASS-21 subscales compared to untreated controls, it would be worthwhile to see whether this rate of improvement can be maintained over a longer period (e.g., 3–6 months). Our study would have benefited from a larger sample size to explore whether a more heterogeneous patient population (with more and less severe anxiety/depressive symptoms) can also benefit from biophysical treatment. To address these limitations, we are currently undertaking and finalizing a larger controlled trial including approximately 100 patients with a follow up of three months. Additional mechanistic studies will also help explore potential underlying mechanisms to explain these observed benefits.

## 4. Materials and Methods

### 4.1. Patient Recruitment and Study Design

This was a randomized controlled trial (the protocol was not registered for this trial) and included patients consisting in workers (sales representatives) presenting with mild anxiety symptoms and related symptoms of depression and stress. Patients attending the Studio Medico Gunè, Acerra, Napoli, Italy from January 2018 to June 2018 completed a Generalized Anxiety Disorder 7-item scale (GADS-7) [59] prior to a medical consultation.

Inclusion criteria were: A score > 5 for the GADS-7 [59]; absence of mental disorders, absence of previous diagnosis of major depressive or major anxiety disorders, absence of use of psychotropic drugs and aged between 18 and 70. Exclusion criteria were: The presence of severe psychiatric disorders; cognitive disorders such as overt dementia; drug or alcohol abuse; suicide attempts or current pregnancy.

The research protocol was proposed to patients who met the inclusion criteria, with an explanation of the aims of the study and declaring the possibility that they are assigned by random allocation to the experimental group (biophysical therapy) or the control (placebo) group. Randomization was performed through the website http://www.randomization.com. All patients provided a signed written informed consent form and this study was performed in accordance with the declaration of Helsinki. Psychological/psychiatric assessment (using self-reported DASS-21 questionnaire) and SAA collection was performed at baseline (before the first session of biophysical treatment) and again at the end of treatment (after one month).

### 4.2. Biophysical Therapy

A single biophysical therapy session (MedSelect 729, Wegamed, Germany) including the electromagnetic information transfer through aqueous system procedure (Nomabit Base, Named, Italy) [58], with self-administration once a day for one month, was used as previously described [20,22]. Briefly, this biophysical strategy employed an electromagnetic information transfer via an aqueous system protocol [58] and biomedical wave generator (Med Select 729, Wegamed, Essen Germany) with a low frequency range (carrying and a modulating frequency of 1 Hz–20 KHz and 1–20 Hz, respectively) and earth magnetic field intensity of 50 µT. A pattern of endogenous electromagnetic signals were first recoded via two electrodes placed on the patient. These endogenous signals were continuously replayed and transferred back to the patient though an electromagnetic carpet. The patient remained in the supine position on the carpet in order to achieve a systemic effect. Two electromagnetic probes placed at specific sites permit the achievement of local effects. All output signals were recorded simultaneously using a microelement aqueous solution and dropper (Nomabit Base, NAMED, Lesmo, Italy), to permit the self-administration of drops adhering to an incremental weekly schedule [20]. In this study, the pattern of endogenous electromagnetic frequency patterns were recorded through two electrodes placed on the forehead of the patients, the signals were not replayed back to the patients as usual but were only recorded on the Nomabit Base solution through the EMITTAS procedure [58], as in previous studies [60,61]. The carrying frequency was 7 Hz while the modulating frequency was continuously fluctuated between 4 and 10 Hz at a magnetic field intensity of 50 µT for 10 min. The placebo control group followed the same procedure. The control group received a nonrecorded solution as placebo. The biophysical procedure and electromagnetic transfer to the aqueous solution has been previously described in detail elsewhere [58].

### 4.3. Salivary Alpha Amylase Sampling and Measurement

Sampling of SAA was performed at 8 am for all fasted patients at baseline, the same day of the biophysical therapy procedure, and 8 am after one month as endpoint. Participants were asked to wash their mouth prior to saliva collection to remove any food debris. Approximately 1 ml of unstimulated saliva was collected by asking the patient to spit 2–3 times into a small sterile disposable plastic container. SAA was measured using a commercially available chemoimmunoluminiscence assay kit (Cobas integra400 plus, Roche Diagnostics, Risch-Rotkreuz, Switzerland) and analyser (Cobas e411, Roche Diagnostics, Risch-Rotkreuz, Switzerland).

### 4.4. DASS-21 Questionnaire

Every patient was requested to complete the self-rating DASS-21 questionnaire [62,63]. The DASS-21 is a self-administered questionnaire is composed of a set of three self-report scales with the aim to measure the emotional states of depression, anxiety, and stress. The three DASS-21 scales encompass seven different items, divided into subscales. Scores for depression, anxiety, and stress were calculated by summing the scores for the different items. Recommended cut-off values for the three subscales are shown in Supplementary Material Table S1.

### 4.5. Statistical Analysis

Data are presented as mean ± standard deviation (SD) or number (%). Differences in variables between groups at baseline and after one month were compared by Student’s t-test or Chi-squared test for continuous or categorical variables respectively. All tests were two-tailed and a p-value of < 0.05 was considered statistically significant. Statistical analysis was performed using Instat Software (GraphPad, San Diego, CA, USA).

## 5. Conclusions

Results from this pilot study demonstrate that patients presenting with mild anxiety/stress as can experience a significant reduction in these symptoms as early as one month after biophysical therapy. Both SAA levels and subscales for the self-reported questionnaire DASS-21 were significantly improved compared to individuals in the control group.

Future studies are needed to characterise the mechanisms through which the biophysical effect derived from low electromagnetic waves exert these beneficial anxiolytic effects.

## Figures and Tables

**Table 1:** Baseline clinical characteristics of control and biophysical groups.

Characteristic	All (*N* = 24)	Placebo (*N* = 12)	Biophysical (*N* = 12)	*p*-Value
Age (years)	39.2 ± 7.2	38.3 ± 5.6	40.1 ± 8.6	0.54
Female gender, n (%)	20 (83.3)	10 (83.3)	10 (83.3)	1.00
Cigarette smoker, n (%)	11 (45.8)	6 (50)	5 (41.7)	0.68
Education, n (%)				
High school	24 (100)	12 (100)	12 (100)	1.00
University	7 (29.2)	3 (25)	4 (33.3)	0.65
Salivary amylase (U/mL)	111.8 ± 94.5	105.2 ± 106.8	118.4 ± 84.6	0.74
DASS-21 subscales				
Depression	9.6 ± 2.1	9.5 ± 2.1	9.7 ± 2.2	0.85
Anxiety	6.8 ± 1.8	6.8 ± 1.9	6.7 ± 1.9	0.83
Stress	11.6 ± 3.4	11.7 ± 3.4	11.5 ± 3.6	0.91

Data are presented as mean ± standard deviation or number and percent. Cigarette smoker refers to current cigarette smoker. DASS-21 = The Depression, Anxiety, and Stress Scale. Statistical significance is represented by *p*-values for comparison of variables in placebo and biophysical groups.

## Supplementary Materials

The following are available online at https://www.mdpi.com/1422-0067/21/2/415/s1.

## Abbreviations

DASS-21	Depression Anxiety Stress Scale-21
GAD-7	Generalized Anxiety Disorder 7-item scale
SAA	Salivary alpha-amylase
