# Supplementary material for: Pilot Study on the Effect of Biophysical Therapy on Salivary Alpha-Amylase as a Surrogate Measure of Anxiety/Stress: In Search of a Novel Noninvasive Molecular Approach for the Management of Stress
Source: Int J Mol Sci. 2020 Jan 9;21(2):415. doi: 10.3390/ijms21020415 (PMC7014022; doi:10.3390/ijms21020415)
Supplement: Supplementary file 1 [file ijms-21-00415-s001.pdf]

Supplementary Material Table 1. Recommended cut-off scores for conventional severity labels according to Lovibond et al. 1995.

Severity level	Depression	Anxiety	Stress
Normal	0-9	0-7	0-14
Mild	10-13	8-9	15-18
Moderate	14-20	10-14	19-25
Severe	21-27	15-19	26-33
Extremely severe	28+	20+	34+

The 3 DASS-21 scales encompass 7 different items, divided into subscales. The depression scale evaluates dysphoria, hopelessness, devaluation of life, self-deprecation, lack of interest / involvement, anhedonia and inertia. The anxiety scale assesses autonomic arousal, skeletal muscle effects, situational anxiety, and subjective experience of anxious affect. The stress scale is sensitive to levels of chronic non-specific arousal. It assesses difficulty relaxing, nervous arousal, and being easily upset/agitated, irritable/over-reactive and impatient.
